# Supplementary material for: How much time to figure out how to get where? Route planning and subjective stress under time pressure
Source: PLoS One. 2025 Jan 13;20(1):e0316382. doi: 10.1371/journal.pone.0316382 (PMC11729951; doi:10.1371/journal.pone.0316382)
Supplement: S1 Text — (DOCX) [file pone.0316382.s001.docx]

# Supplemental Method

## Route Review

To determine whether participants followed instructions, we manually reviewed the routes traced by the participants. One graduate student and multiple undergraduate students reviewed each route (*N* = 10,639) traced by participants. To facilitate review, all routes were drawn electronically on plots using the x and y coordinates recorded while the mouse or trackpad button was held down during the tracing portion of the task, and overlaid on the corresponding stimulus image. Each route was saved as an image file. Based on the preregistered exclusion criteria, all performance data from any participant with more than five excluded routes would be excluded.

### Procedure One

The graduate student looked at every route image and coded a 0 if the route did not exist, it obviously did not follow the paths, or obviously did not visit one or more of the target locations. Routes for which it appeared that the participant followed instructions were coded as 1. These exclusion codes were saved as a binary variable. Unlike the subsequent procedures, these codes were created in a few days by only one reviewer, who was intimately familiar with the stimulus design and preregistered exclusion criteria.

**Table S1**

*Codes and descriptions used during route review procedure one*

| Code | Meaning |
| --- | --- |
| 0 | Participant did not follow instructions |
| 1 | Participant followed instructions |

*Note.* For option one, the exclusion code is 0.

### Procedure Two

Procedures two and three involved multiple coders, who were systematically trained but relatively naïve to the stimulus design and preregistered exclusion criteria. Review procedures two and three occurred over two semesters.

Three undergraduate students reviewed the 10,639 route images and coded each from one to six. For each route reviewed by all three undergraduates, and each route coded consistently by two undergraduates, the modal code was saved. Some routes were coded by only one undergraduate student and the graduate student reviewed each of those, as well all routes which were only coded by two undergraduates and had inconsistent codes (only the graduate student used codes eight and nine). Based on the subjective criterion of “how much deviation is too much deviation from the paths?”, the single original coder was probably more likely than the subsequent coders to code a route a 4 when deciding whether the route was scored "4 - questionable" or "5 - no question exclusion".

**Table S2**

*Codes and descriptions used during route review procedure two*

| Code | Meaning |
| --- | --- |
| 1 | Participant followed all instructions |
| 2 | Participant did not go to the waypoint (for trials that don't return to the start) |
| 3 | Participant did not go to the waypoint (for trials that return to the start) |
| 4 | Participant did not try to follow the paths (questionable) |
| 5 | Participant did not try to follow the paths (no question) |
| 6 | Participant did not trace a route from start to finish |
| 8 | Five or more waypoints missed so participant will be excluded |
| 9 | Five or more DNF instructions so participant will be excluded |

*Note.* For option two, exclusion codes are 2, 3, 5, 6, 8, and 9.

### Procedure Three

Seven undergraduates (the same three as previous plus four more) reviewed all the routes that were previously coded 4 or 5, as well as all routes for which there were previously inconsistent codes between the raters (*N* = 2,738). The modal rating was taken for all but *n* = 19, which did not have a single modal rating. For those 19, the graduate student who conducted the first procedure reviewed each and decided between the modal options.

After Procedure Two, we realized that it would be helpful to clarify the distinction between codes 4 and 5. The team drafted consensus guidelines as reference materials to inform decisions about routes which deviated from the path but could have been planned in good faith, to facilitate objectivity during review. For example, there were places on the maps which were difficult or impossible to determine whether a route was valid or existed at all. If we thought that a participant could be following instructions in good faith and still make a given error, we decided to include the route in analyses per preregistered criteria. These potential errors were unique to each stimulus. Annotated stimuli were used as a reference and are available with the study materials on OSF (stimulus_slides_marked_up_with_notes.pptx).

**Table S3**

*Codes and descriptions used during route review procedure three*

| Code | Meaning  Detail Provided in Coding Instructions |
| --- | --- |
| 1 | Participant followed all instructions |
|  | Participant didn’t make any mistakes (as outlined below) and the only deviation (if any) is that the route drawn is somewhat off of the path |
|  |  |
| 2 | Participant did not go to the waypoint |
|  | For trials that don't return to the start |
|  |  |
| 3 | Participant did not go to the waypoint |
|  | For trials that return to the start. Includes if a participant draws a line directly from the start to finish (usually a really short segment). |
|  |  |
| 4 | Participant makes one of the mistakes noted on the marked up stimuli |
|  | If in the marked-up slides it says "this is ok" or "there is a path here", etc., then code it as a 1. |
|  |  |
| 5 | Participant did not try to follow the paths (no question) |
|  | Participant either blatantly disregards the paths or makes a major cut across an obvious park, building, or river. Also includes if the participant goes into the border to connect two roads |
|  |  |
| 6 | Participant did not trace a route from start to finish |
|  | Also includes if the participant draws a loop from the finish back to the start (effectively doubling the route length) or from the start to the finish then to the waypoint |

*Note.* The variable is labelled codeInstructions and the purpose of the variable is to answer the question, “Did the participant follow the instructions and the paths?”. Stimuli numbers 42-85 could receive a 2 and stimuli numbers 86-120 could receive a 3. Excluded codes were 2, 3, 5, and 6.

## Materials

### Map Stimuli

The latitude and longitude in decimal degrees for each map stimulus are available in the S1_Map_Coordinates.csv. There are coordinates for the center of each map stimulus and the center of each small mark used as a starting point, ending point, or waypoint (yellow star, red octagon, and purple triangle, respectively). We also included approximate coordinates for the upper right and bottom left corner of each map; these corner coordinates can be used to recreate the maps at the original scale in Google Maps or another mapping software.

### Engagement, Distress, and Worry

We administered the Short Stress State Questionnaire [1] to measure engagement, distress, and worry at two time points (before and after the task). The baseline questions referred to “at the moment” for emotion words and “during the past thirty minutes” for short phrases; the post-task questions referred to “during the task” and “while performing the task”, respectively. Response options to all items were on a five-point Likert-style scale from 1, *Not at all,* to 5, *Extremely*.

Engagement (*α_pre_* = .81, *α_post_* = .87) was assessed by seven items, a mix of emotion words (e.g., “Active”) and phrases (e.g., “I am/was committed to attaining my performance goals”). Distress (*α_pre_* = .85, *α_post_* = .81) was assessed by seven emotion word items (e.g., “Impatient”, “Angry”, “Depressed”). Worry (*α_pre_* = .84, *α_post_* = .85) was assessed by seven phrases (e.g., “I feel/felt self-conscious”, “I thought about how others have done on this task”).

### Ideal Route Length

Maps varied with respect to how long the route would need to be given the location of the start, waypoint, if applicable, and end point. Some participants and some trials within participants, by random chance, presented maps with longer ideal routes. Other stimuli had shorter ideal routes; models used for confirmatory hypothesis testing do not explicitly account for this variability in trial-level outcomes. We chose to explore one potential operationalization of ideal route length, "as-the-crow-flies”, without respect to the rule of the task to stay on roads. From this perspective, the ideal route length is the combined length of the straight lines connecting the start, waypoint (if present), and endpoint. We added two ideal route length predictors in exploratory models. One variable captured variation between participants (grand-mean centered and *z*-transformed) and the other captured variation within participants (person-mean centered and *z*-transformed).

### Financial Performance Motivation

One of the assumptions of the experimental manipulations was that participants were motivated to complete as many routes as possible within the allocated time due to the monetary performance bonus. Participants reported financial motivation after completing the manipulation check by responding to the question “How much were you motivated by the performance bonus money?” on a 5-point Likert-style scale from 1, *Not at all*, to 5, *Extremely*.

### Age

Participants reported their age in years.

### Other Measures Administered

Five Facet Mindfulness Questionnaire [2]

Multi-State Mindfulness Questionnaire [3]

Performance Satisfaction (single item)

Subjective Performance Estimates and Confidence in Estimates:

Number of maps completed

Average number of seconds to complete each map

Average waiting period length in seconds

Total waiting time (in 30-second increments from 0 minutes 30 seconds to 20 minutes or more)

Open ended emotions during the waiting periods

Did you feel any emotions during the waiting periods? (For example, did you feel bored, impatient, anxious, or frustrated?) If so, please describe them in the box below:

Open ended emotion regulation during the waiting periods

Did you do anything to manage your emotions during the waiting periods? (For example, did you tap your fingers, check your phone, or take a sip of a drink?) If so, please use the box below to describe what you did (remember, there are no wrong answers):

Santa Barbara Sense of Direction [4]

Trait Inventory of Cognitive and Somatic Anxiety [5,6] (Preliminary analyses have been presented at a conference.)

Multi-family Emotion Regulation Questionnaire (unpublished) (Preliminary analyses have been presented at a conference.)

Decision Difficulty subscale of Maximization Inventory [7]

Experience with maps (single item)

Experience with computers (single item)

Experiences with meditation and yoga

Multi-source Interference Task [8]

Demographics (Education, Single-item political Ideology)

# Exploratory Analyses

We conducted exploratory analyses to better understand participants’ experience of stress and how it relates to performance during the task. Specifically, we examined zero-order correlations between the task metrics, age, financial motivation, and engagement, distress, and worry at baseline and post-task (Helton & Näswall, 2015). We also re-fit models accounting for interaction device (trackpad or mouse), type of map stimuli (no waypoint, waypoint with different start and end points, waypoint with same start and end points), and ideal route length.

## Engagement, Distress, and Worry

As shown in Figure S1, we found that engagement, *M_diff_* = 0.33, CI_95%_ = [0.26, 0.40], *t* = 9.45, *p* < .001, and distress, *M_diff_* = 0.12, CI_95%_ = [0.04, 0.20], *t* = 2.85, *p* = .005, were higher post-task than at baseline. By contrast, worry was lower at post-task than at baseline, *M_diff_* = -0.26, CI_95%_ = [-0.36, -0.17], *t* = -5.29, *p* < .001). Correlations with other measures are shown in Table S4.

**Fig S1. Engagement, Distress, and Worry at Baseline and Post-Task**

Figure displays participant-level data. The legend labels, from top to bottom, correspond with the violin plots from left to right. For each violin (i.e., combination of type of subjective stress state and measurement time), the horizontal lines indicate quartiles and the dot indicates the mean.

**Table S4**

*Zero-order correlations between individual-level variables*

| Variable | *M* | *SD* | 1 | 2 | 3 | 4 | 5 | 6 | 7 | 8 | 9 | 10 | 11 | 12 | 13 | 14 |
| --- | --- | --- | --- | --- | --- | --- | --- | --- | --- | --- | --- | --- | --- | --- | --- | --- |
| 1. Subjective Time Pressure | 50.42 | 26.58 |  |  |  |  |  |  |  |  |  |  |  |  |  |  |
| 2. Avg Trial Stress | 37.35 | 22.30 | **.76^**^** |  |  |  |  |  |  |  |  |  |  |  |  |  |
| 3. Avg Plan Time | 10.57 | 6.38 | -.04 | .02 |  |  |  |  |  |  |  |  |  |  |  |  |
| 4. Avg Route Length | 1.86 | 0.25 | .11 | **.17^*^** | **.28^**^** |  |  |  |  |  |  |  |  |  |  |  |
| 5. Number of Routes | 30.12 | 16.24 | -.05 | **-.23^**^** | **-.58^**^** | **-.30^**^** |  |  |  |  |  |  |  |  |  |  |
| 6. Base Subjective Stress | 33.27 | 24.59 | **.51^**^** | **.58^**^** | -.08 | -.01 | -.06 |  |  |  |  |  |  |  |  |  |
| 7. Post Subjective Stress | 46.50 | 27.70 | **.76^**^** | **.88^**^** | -.02 | **.15^*^** | **-.13^*^** | **.56^**^** |  |  |  |  |  |  |  |  |
| 8. Base Engage | 3.69 | 0.61 | -.13 | **-.19^**^** | .13 | .10 | -.07 | **-.27^**^** | **-.15^*^** |  |  |  |  |  |  |  |
| 9. Post Engage | 4.02 | 0.55 | -.10 | **-.17^**^** | .13 | -.04 | .05 | **-.14^*^** | **-.16^*^** | **.59^**^** |  |  |  |  |  |  |
| 10. Base Distress | 1.48 | 0.50 | .13 | **.21^**^** | **-.13*** | -.08 | .05 | **.32^**^** | **.20^**^** | **-.40^**^** | **-.30^**^** |  |  |  |  |  |
| 11. Post Distress | 1.60 | 0.63 | **.32^**^** | **.34^**^** | **-.18^**^** | .12 | -.01 | **.25^**^** | **.34^**^** | **-.33^**^** | **-.50^**^** | **.43^**^** |  |  |  |  |
| 12. Base Worry | 2.18 | 0.84 | **.21^**^** | **.25^**^** | **-.20^**^** | .05 | .01 | **.28^**^** | **.27^**^** | **-.19^**^** | -.04 | **.40^**^** | **.19^**^** |  |  |  |
| 13. Post Worry | 1.91 | 0.80 | **.31^**^** | **.35^**^** | -.10 | **.19^**^** | -.11 | **.23^**^** | **.34^**^** | -.00 | -.12 | **.21^**^** | **.27^**^** | **.58^**^** |  |  |
| 14. Financial Motivation | 3.51 | 1.18 | **.19^**^** | **.13^*^** | **-.15^*^** | -.03 | .13 | .11 | **.17^*^** | **.16^*^** | **.34^**^** | -.01 | -.06 | **.26^**^** | **.24^**^** |  |
| 15. Age | 42.35 | 16.78 | **-.14^*^** | -.05 | **.41^**^** | .07 | **-.33^**^** | **-.14^*^** | -.09 | **.26^**^** | **.16^*^** | **-.30^**^** | **-.19^**^** | **-.44^**^** | **-.26^**^** | **-.32^**^** |

Subjective Time Pressure = one item about the whole task, Avg = average of trial-level responses, Number of Routes = number of routes completed successfully, Base = score at baseline, Post = score at post-task. *M* is mean and *SD* is standard deviation. **Bold** *p*-values indicate statistical significance

; * indicates p < .05. ** indicates p < .01.

We examined models that contained all three subjective stress states as simultaneous predictors of trial-level stress and performance outcomes. Increased trial-level stress was associated with greater post-task distress (*b* = 6.94, CI_95%_ = [1.76, 12.12], *p* = .009) and worry (*b* = 8.44, CI_95%_ = [4.17, 12.70], *p* < .001). Higher baseline worry was associated with shorter planning times (*OR* = 0.86, CI_95%_ = [0.80, 0.93], *p* < .001). Pre-task distress was associated with shorter routes (*b* = -0.08, CI_95%_ = [-0.15, -0.02], *p* = .016) and inversely, post-task distress was associated with longer routes (*b* = 0.07, CI_95%_ = [0.02, -0.13], *p* = .008).^[[1]](#footnote-1)^

## Interaction Device and Map Type

We examined the primary models from confirmatory testing (H1-H5), with the type of interaction device and the type of map included as covariates (Tables S5-S6). Interaction device was sum to zero contrast coded with mouse and trackpad (-0.5, 0.5); the regression estimate is the difference between the device types. Map type was treatment contrast coded; each regression estimate compares one of the map types with a waypoint to the map type with no waypoint. When subjective stress was included as a fixed-effect predictor (exploring H4 and H5), trackpad use predicted less planning time and nominally, but not statistically significantly shorter routes. Maps with a waypoint and different start and end points were associated with increased subjective stress, compared to maps without waypoints. Maps with a waypoint and the same start and end points were associated with less planning time, compared to maps without a waypoint. Both map types with waypoints were associated with longer tracing time. Map type was not statistically significantly associated with route length in these models with ideal route length, but both types of maps with waypoints were nominally associated with longer routes.

## Ideal Route Length

The exploratory models that we report in Tables S5-S6 also include the ideal route length, as the crow flies. Two variables were calculated, which represent the average ideal route length for each participant, as well as the deviation from that person’s average on any given trial. Participants took more time to plan their route when they saw a map with a longer ideal route than when they saw a map with a shorter ideal route. Also, participants who saw maps with longer ideal routes on average traced longer routes than participants who saw maps with shorter ideal routes. Nominally, but not statistically significantly, participants also traced longer routes on maps with longer ideal routes than on maps with shorter ideal routes.

**Table S5**

*Exploratory Models with Device Type, Map Type, and Ideal Length*

| **Outcome** | **Subjective stress** | | | | **Plan time** | | | | **Trace time** | | | | **Route length** | | | |
| --- | --- | --- | --- | --- | --- | --- | --- | --- | --- | --- | --- | --- | --- | --- | --- | --- |
| *Fixed Effect Predictors* | *b* | *SE* | *t* | *p* | *OR* | *SE* | *t* | *p* | *OR* | *SE* | *t* | *p* | *b* | *SE* | *t* | *p* |
| Intercept | 36.25 | 1.76 | 20.65 | **<.001** | 8.52 | 0.34 | 53.44 | **<.001** | 27.60 | 2.10 | 43.62 | **<.001** | 1.79 | 0.06 | 31.83 | **<.001** |
| Less - More Time | 3.96 | 2.99 | 1.32 | .186 | 1.07 | 0.05 | 1.35 | .178 | 1.04 | 0.08 | 0.49 | .622 | 0.01 | 0.03 | 0.41 | .681 |
| Long - Short Waits | -0.41 | 2.97 | -0.14 | .891 | 1.11 | 0.06 | 2.13 | **.034** | 1.11 | 0.09 | 1.35 | .177 | 0.01 | 0.03 | 0.27 | .791 |
| Urgent - Not Urgent | 8.69 | 0.77 | 11.33 | **<.001** | 0.88 | 0.02 | -6.78 | **<.001** | 0.92 | 0.01 | -5.88 | **<.001** | -0.00 | 0.01 | -0.33 | .738 |
| Trackpad - Mouse | 3.72 | 3.02 | 1.23 | .218 | 0.85 | 0.04 | -3.15 | **.002** | 1.06 | 0.09 | 0.75 | .456 | -0.05 | 0.03 | -1.72 | .086 |
| Waypoint Different End -  No Waypoint | 2.84 | 1.09 | 2.61 | **.009** | 1.02 | 0.04 | 0.60 | .549 | 1.31 | 0.10 | 3.53 | **<.001** | 0.07 | 0.07 | 1.03 | .305 |
| Waypoint Same End -  No Waypoint | 2.11 | 1.52 | 1.38 | .167 | 0.89 | 0.05 | -2.04 | **.042** | 1.25 | 0.13 | 2.05 | **.041** | 0.10 | 0.10 | 1.07 | .286 |
| Ideal Route Length (PMC) | -7.50 | 7.53 | -1.00 | .319 | 1.36 | 0.18 | 2.35 | **.019** | 1.14 | 0.21 | 0.72 | .471 | 0.13 | 0.08 | 1.74 | .082 |
| Ideal Route Length (GMC) | 9.49 | 7.64 | 1.24 | .214 | 0.78 | 0.10 | -1.86 | .062 | 1.04 | 0.20 | 0.22 | .822 | 0.36 | 0.09 | 4.23 | **<.001** |
| Time × Waits | 1.54 | 5.95 | 0.26 | .796 | 0.96 | 0.10 | -0.38 | .707 | 1.08 | 0.17 | 0.53 | .596 | 0.07 | 0.06 | 1.23 | .220 |
| Time × Urgency | -0.05 | 1.53 | -0.03 | .973 | 1.01 | 0.04 | 0.26 | .793 | 1.01 | 0.03 | 0.41 | .684 | 0.01 | 0.02 | 0.57 | .570 |
| Waits × Urgency | 2.54 | 1.53 | 1.66 | .098 | 0.99 | 0.04 | -0.30 | .761 | 1.00 | 0.03 | 0.08 | .935 | -0.02 | 0.02 | -1.03 | .302 |
| Time × Waits × Urgency | 3.70 | 3.07 | 1.21 | .228 | 1.00 | 0.08 | -0.03 | .977 | 1.05 | 0.06 | 0.80 | .422 | -0.02 | 0.04 | -0.63 | .527 |
| *Random Effects Clusters* | | | | | | | | | | | | | | | | |
| τ_00_ | 453.05 _ID_ | | | | 0.14 _ID_ | | | | 0.05 _ID_ | | | | 0.04 _ID_ | | | |
|  | 9.57 _mapID_ | | | | 0.01 _mapID_ | | | | 0.01 _mapID_ | | | | 0.05 _mapID_ | | | |
| τ_11_ | 101.83 _ID.urgMsgurgent_ | | | | 0.05 _ID.urgMsgurgent_ | | | | 0.01 _ID.urgMsgurgent_ | | | | 0.00 _ID.urgMsgurgent_ | | | |
| ρ_01_ | 0.05 _ID_ | | | | -0.29 _ID_ | | | | -0.29 _ID_ | | | | -0.31 _ID_ | | | |
| ICC _ID_ | .62 | | | | .32 | | | | .34 | | | | .21 | | | |
| ICC _urgMsg \| ID_ | .14 | | | | .10 | | | | .09 | | | | .01 | | | |
| ICC _map_ | .01 | | | | .03 | | | | .08 | | | | .25 | | | |
| N | 120 _mapID_ | | | | 120 _mapID_ | | | | 120 _mapID_ | | | | 120 _mapID_ | | | |
|  | 226 _ID_ | | | | 226 _ID_ | | | | 226 _ID_ | | | | 226 _ID_ | | | |
| Observations | 6709 | | | | 6757 | | | | 6757 | | | | 6757 | | | |
| Marginal R^2^ (predictors) | .055 | | | | .050 | | | | .357 | | | | .586 | | | |
| Conditional R^2^ | .750 | | | | .421 | | | | .650 | | | | .780 | | | |

Intercepts for subjective stress and route length represent the predicted value of the outcome, and the intercept for plan time is the odds ratio, when all experimental manipulations are held constant at the average and device use is held constant at the average, for maps with no waypoint. For models with subjective stress as a predictor (rightmost columns), subjective stress is held constant at the grand mean and the mean for each participant. ID specifies the random intercept for participant, map the random intercept for map, and urgMsg | ID is the random slope for urgency by participant. GMC is grand mean-centered, which indicates between-persons variability in subjective stress. PMC is person mean-centered, which indicates within-person variability in subjective stress. **Bold** *p*-values indicate statistical significance.

**Table S6**

*Exploratory Models with Subjective Stress, Device Type, Map Type, and Ideal Length*

| **Outcome** | **Plan time** | | | | **Trace time** | | | | **Route length** | | | |
| --- | --- | --- | --- | --- | --- | --- | --- | --- | --- | --- | --- | --- |
| *Fixed Effect Predictors* | *OR* | *SE* | *t* | *p* | *OR* | *SE* | *t* | *p* | *b* | *SE* | *t* | *p* |
| Intercept | 8.55 | 0.34 | 53.87 | **<.001** | 25.74 | 0.78 | 107.47 | **<.001** | 1.80 | 0.05 | 32.99 | **<.001** |
| Less - More Time | 1.06 | 0.05 | 1.18 | .240 | 1.01 | 0.03 | 0.37 | .713 | 0.00 | 0.03 | 0.17 | .862 |
| Long - Short Waits | 1.11 | 0.06 | 2.15 | **.031** | 1.10 | 0.03 | 3.22 | **.001** | 0.01 | 0.03 | 0.29 | .769 |
| Urgent - Not Urgent | 0.86 | 0.02 | -7.46 | **<.001** | 0.89 | 0.01 | -10.07 | **<.001** | -0.05 | 0.01 | -4.83 | **<.001** |
| Subjective Stress PMC | 1.002 | 0.00 | 4.52 | **<.001** | 1.004 | 0.00 | 16.85 | **<.001** | 0.005 | 0.00 | 17.53 | **<.001** |
| Subjective Stress GMC | 1.002 | 0.00 | 1.67 | .094 | 1.005 | 0.00 | 6.62 | **<.001** | 0.002 | 0.00 | 2.68 | **.007** |
| Trackpad - Mouse | 0.84 | 0.04 | -3.30 | **.001** | 0.99 | 0.03 | -0.20 | .842 | -0.06 | 0.03 | -2.00 | **.046** |
| Waypoint Different End -  No Waypoint | 1.02 | 0.04 | 0.47 | .638 | 1.29 | 0.04 | 7.64 | **<.001** | 0.06 | 0.07 | 0.86 | .391 |
| Waypoint Same End -  No Waypoint | 0.89 | 0.05 | -2.14 | **.032** | 1.23 | 0.06 | 4.42 | **<.001** | 0.09 | 0.09 | 0.99 | .321 |
| Ideal Route Length (PMC) | 1.38 | 0.18 | 2.45 | **.014** | 1.29 | 0.10 | 3.23 | **.001** | 0.14 | 0.07 | 1.90 | .058 |
| Ideal Route Length (GMC) | 0.76 | 0.10 | -2.00 | **.046** | 0.92 | 0.07 | -1.08 | .281 | 0.34 | 0.08 | 4.05 | **<.001** |
| Time × Waits | 0.96 | 0.10 | -0.41 | .685 | 1.11 | 0.07 | 1.70 | .090 | 0.07 | 0.06 | 1.19 | .235 |
| Time × Urgency | 1.01 | 0.04 | 0.32 | .749 | 1.01 | 0.02 | 0.42 | .676 | 0.01 | 0.02 | 0.51 | .613 |
| Waits × Urgency | 0.98 | 0.04 | -0.50 | .618 | 0.99 | 0.02 | -0.35 | .727 | -0.03 | 0.02 | -1.58 | .114 |
| Time × Waits × Urgency | 0.99 | 0.08 | -0.12 | .904 | 1.02 | 0.05 | 0.52 | .601 | -0.04 | 0.04 | -0.94 | .346 |
| *Random Effects Clusters* | | | | | | | | | | | | |
| τ_00_ | 0.14 _ID_ | | | | 0.05 _ID_ | | | | 0.04 _ID_ | | | |
|  | 0.01 _mapID_ | | | | 0.01 _mapID_ | | | | 0.05 _mapID_ | | | |
| τ_11_ | 0.05 _ID.urgMsgurgent_ | | | | 0.02 _ID.urgMsgurgent_ | | | | 0.00 _ID.urgMsgurgent_ | | | |
| ρ_01_ | -0.30 _ID_ | | | | -0.29 _ID_ | | | | -0.32 _ID_ | | | |
| ICC _ID_ | .32 | | | | .34 | | | | .22 | | | |
| ICC _urgMsg \| ID_ | .11 | | | | .11 | | | | .03 | | | |
| ICC _map_ | .03 | | | | .07 | | | | .24 | | | |
| N | 120 _mapID_ | | | | 120 _mapID_ | | | | 120 _mapID_ | | | |
|  | 226 _ID_ | | | | 226 _ID_ | | | | 226 _ID_ | | | |
| Observations | 6709 | | | | 6709 | | | | 6709 | | | |
| Marginal R^2^ (predictors) | .055 | | | | .400 | | | | .601 | | | |
| Conditional R^2^ | .425 | | | | .675 | | | | .789 | | | |

Intercepts for subjective stress and route length represent the predicted value of the outcome, and the intercept for plan time is the odds ratio, when all experimental manipulations are held constant at the average and trackpad use is held constant at the average, for maps with no waypoint. For models with subjective stress as a predictor (rightmost columns), subjective stress is held constant at the grand mean and the mean for each participant. ID specifies the random intercept for participant, map the random intercept for map, and urgMsg | ID is the random slope for urgency by participant. GMC is grand mean-centered, which indicates between-persons variability in subjective stress. PMC is person mean-centered, which indicates within-person variability in subjective stress. **Bold** *p*-values indicate statistical significance.

# Exploratory Discussion

In this Supplemental, we explored individual differences, including three qualitatively different stress states at baseline and post-task and also explored interaction device, map type, and ideal route length as covariates in the models used for confirmatory hypothesis testing. The interpretation of the models used for the confirmatory hypothesis testing did not change based on the exploratory results.

Trackpad, compared to mouse use was associated with reduced planning time and when controlling for subjective stress, reduced route length. Participants reported more subjective stress for maps with waypoints. When there was a waypoint with the same start and end points, plan time was shorter and when ideal route length was longer, plan time was also longer. Both types of maps with waypoints, as well as maps with longer ideal routes were associated with longer tracing times. Only the average ideal route length for the participant was a statistically significant predictor of increased route length, but the maps with waypoints and maps with longer ideal route lengths nominally predicted increased route length.

Results suggest directions for future research into the experiences of stress during navigation, such as zero-order correlations between financial motivation and more subjective stress, or between age and greater engagement, less distress, and less worry (Table S4). In line with research on stress states during another type of navigational task [e.g., orienteering, 1], engagement on average increased from before to after the task, while worry decreased. However, unlike Helton and Näswall’s (2015) study of orienteering, distress increased in this study.

References

1. Helton WS, Näswall K. Short Stress State Questionnaire: Factor structure and state change assessment. Eur J Psychol Assess. 2015 Jun 1;31(1):20–30.

2. Baer RA, Smith GT, Hopkins J, Krietemeyer J, Toney L. Using self-report assessment methods to explore facets of mindfulness. Assessment. 2006 Mar;13(1):27–45.

3. Blanke ES, Brose A. Mindfulness in daily life: A multidimensional approach. Mindfulness. 2017 Jun;8(3):737–50.

4. Hegarty M, Richardson AE, Montello DR, Lovelace K, Subbiah I. Development of a self-report measure of environmental spatial ability. Intelligence. 2002 Sep 1;30(5):425–47.

5. Grös DF, Antony MM, Simms LJ, McCabe RE. Psychometric properties of the State-Trait Inventory for Cognitive and Somatic Anxiety (STICSA): Comparison to the State-Trait Anxiety Inventory (STAI). Psychol Assess. 2007;19(4):369–81.

6. Ree MJ, French D, MacLeod C, Locke V. Distinguishing cognitive and somatic dimensions of state and trait anxiety: Development and validation of the State-Trait Inventory for Cognitive and Somatic Anxiety (STICSA). Behav Cogn Psychother [Internet]. 2008 May [cited 2021 Mar 9];36(03). Available from: http://www.journals.cambridge.org/abstract_S1352465808004232

7. Turner BM, Rim HB, Betz NE, Nygren TE. The Maximization Inventory. Judgm Decis Mak. 2012;7(1):14.

8. Bush G, Shin LM. The Multi-Source Interference Task: an fMRI task that reliably activates the cingulo-frontal-parietal cognitive/attention network. Nat Protoc. 2006 Jun;1(1):308–13.

1. Exploratory interactions are not reported in this Supplemental Material. [↑](#footnote-ref-1)
