# Supplementary figures and images for: How much time to figure out how to get where? Route planning and subjective stress under time pressure
Source: PLoS One. 2025 Jan 13;20(1):e0316382. doi: 10.1371/journal.pone.0316382 (PMC11729951; doi:10.1371/journal.pone.0316382)

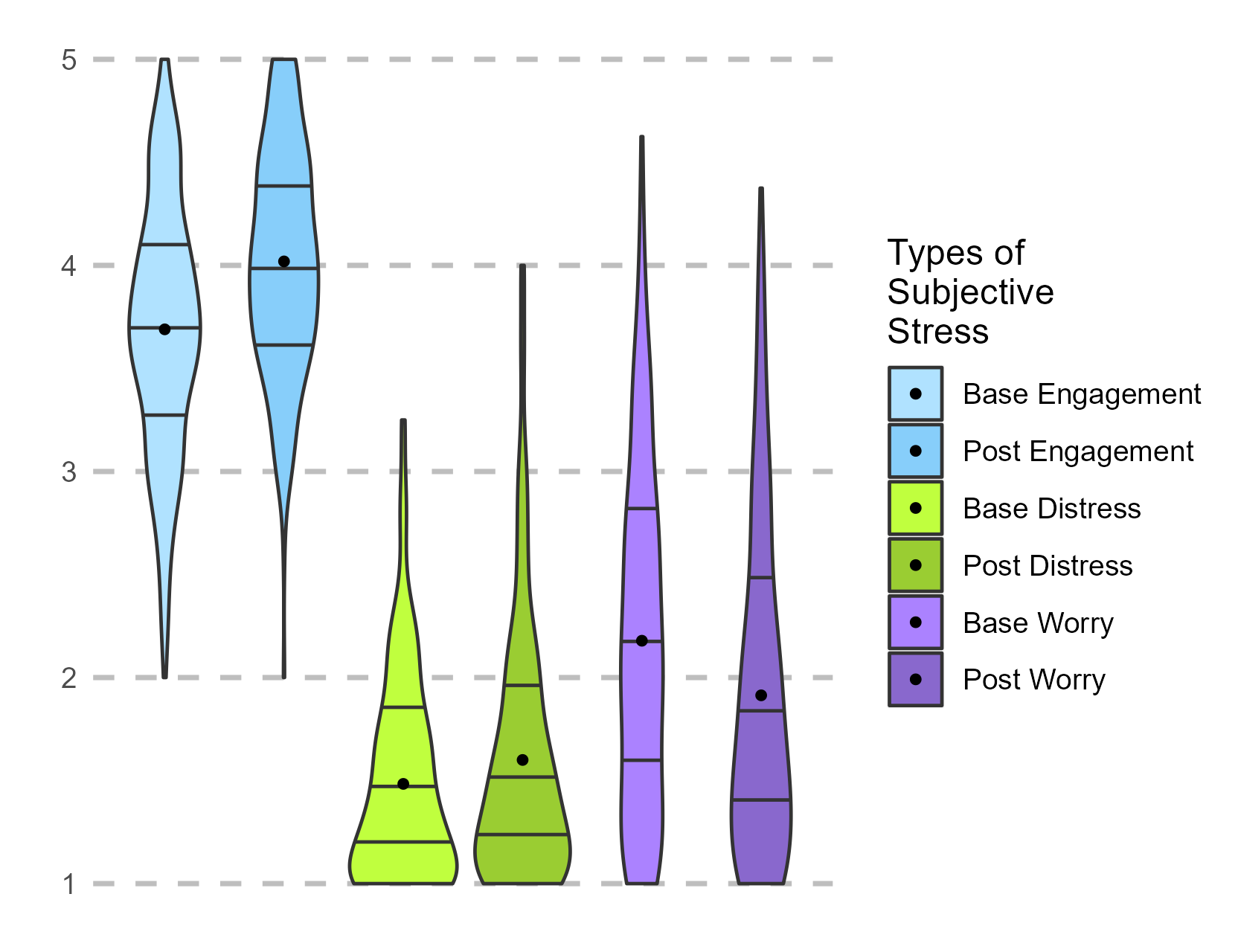

Supplement: S1 Fig — Figure displays participant-level data. The legend labels, from top to bottom, correspond with the violin plots from left to right. For each violin (i.e., combination of type of subjective stress state and measurement time), the horizontal lines indicate quartiles and the dot indicates the mean. (TIF) [file pone.0316382.s002.tif]
