# Supplementary material for: How much time to figure out how to get where? Route planning and subjective stress under time pressure
Source: PLoS One. 2025 Jan 13;20(1):e0316382. doi: 10.1371/journal.pone.0316382 (PMC11729951; doi:10.1371/journal.pone.0316382)
Supplement: Attachment — Submitted filename: Response to Reviewers.docx [file pone.0316382.s004.docx]

Journal Requirements:

When submitting your revision, we need you to address these additional requirements.

1. Please ensure that your manuscript meets PLOS ONE's style requirements, including those for file naming. The PLOS ONE style templates can be found at

https://journals.plos.org/plosone/s/file?id=wjVg/PLOSOne_formatting_sample_main_body.pdf and 
https://journals.plos.org/plosone/s/file?id=ba62/PLOSOne_formatting_sample_title_authors_affiliations.pdf

The manuscript and manuscript files have been formatted according to the requirements above.

2. We note that the grant information you provided in the ‘Funding Information’ and ‘Financial Disclosure’ sections do not match.

When you resubmit, please ensure that you provide the correct grant numbers for the awards you received for your study in the ‘Funding Information’ section.

This research was completed under a cooperative agreement, as described in the Cover Letter and the submission portal. Although there was not a grant to a specific researcher, Heather Urry (HLU) can be identified as the recipient for ease of reporting.

3. Please note that funding information should not appear in the Acknowledgments section or other areas of your manuscript. We will only publish funding information present in the Funding Statement section of the online submission form. Please remove any funding-related text from the manuscript.

4. Please ensure that you refer to Figure 7 in your text as, if accepted, production will need this reference to link the reader to the figure.

We revised the figures and referred to each figure in the text.

5. Please upload a new copy of Figures 1-3 as the detail is not clear. Please follow the link for more information:

https://blogs.plos.org/plos/2019/06/looking-good-tips-for-creating-your-plos-figures-graphics/
https://blogs.plos.org/plos/2019/06/looking-good-tips-for-creating-your-plos-figures-graphics/

We revised the figures and reformatted them according to the specifications above.

6. We note that Figures 1-3 in your submission contain map images which may be copyrighted. All PLOS content is published under the Creative Commons Attribution License (CC BY 4.0), which means that the manuscript, images, and Supporting Information files will be freely available online, and any third party is permitted to access, download, copy, distribute, and use these materials in any way, even commercially, with proper attribution. For these reasons, we cannot publish previously copyrighted maps or satellite images created using proprietary data, such as Google software (Google Maps, Street View, and Earth). For more information, see our copyright guidelines: http://journals.plos.org/plosone/s/licenses-and-copyright.
 
We require you to either (1) present written permission from the copyright holder to publish these figures specifically under the CC BY 4.0 license, or (2) remove the figures from your submission:
 
1) You may seek permission from the original copyright holder of Figures 1-3 to publish the content specifically under the CC BY 4.0 license.

We cannot obtain an exception to the Google Maps policy for these figures, so we have proceeded with option 2) below.
 
We recommend that you contact the original copyright holder with the Content Permission Form (http://journals.plos.org/plosone/s/file?id=7c09/content-permission-form.pdf) and the following text:
“I request permission for the open-access journal PLOS ONE to publish XXX under the Creative Commons Attribution License (CCAL) CC BY 4.0 (http://creativecommons.org/licenses/by/4.0/). Please be aware that this license allows unrestricted use and distribution, even commercially, by third parties. Please reply and provide explicit written permission to publish XXX under a CC BY license and complete the attached form.”
 
Please upload the completed Content Permission Form or other proof of granted permissions as an ""Other"" file with your submission.
 
In the figure caption of the copyrighted figure, please include the following text: “Reprinted from [ref] under a CC BY license, with permission from [name of publisher], original copyright [original copyright year].”
 
2) If you are unable to obtain permission from the original copyright holder to publish these figures under the CC BY 4.0 license or if the copyright holder’s requirements are incompatible with the CC BY 4.0 license, please either i) remove the figure or ii) supply a replacement figure that complies with the CC BY 4.0 license. Please check copyright information on all replacement figures and update the figure caption with source information. If applicable, please specify in the figure caption text when a figure is similar but not identical to the original image and is therefore for illustrative purposes only.

We chose to remove the copyrighted content from the publication and did not create replacement content. Instead, we have retained the most relevant details and referred to content that is available for fair use on OSF.

The following resources for replacing copyrighted map figures may be helpful:
 
USGS National Map Viewer (public domain): http://viewer.nationalmap.gov/viewer/
The Gateway to Astronaut Photography of Earth (public domain): http://eol.jsc.nasa.gov/sseop/clickmap/
Maps at the CIA (public domain): https://www.cia.gov/library/publications/the-world-factbook/index.html and https://www.cia.gov/library/publications/cia-maps-publications/index.html
NASA Earth Observatory (public domain): http://earthobservatory.nasa.gov/
Landsat: http://landsat.visibleearth.nasa.gov/
USGS EROS (Earth Resources Observatory and Science (EROS) Center) (public domain): http://eros.usgs.gov/#
Natural Earth (public domain): http://www.naturalearthdata.com/

7. Please include captions for your Supporting Information files at the end of your manuscript, and update any in-text citations to match accordingly. Please see our Supporting Information guidelines for more information: http://journals.plos.org/plosone/s/supporting-information.

Captions for the three supplemental materials have been added at the end of the manuscript.

**Reviewer #1:**

This is a well written paper which addresses and interesting question and render some interesting results. Having said this, I have a couple of major concerns which I will outline below. I believe that these concerns need to be addressed before the paper can be published.

Thank you. We believe that we have addressed each of the concerns in the revised manuscript.

Major concerns:
The authors state that the urgency manipulation affect planning time and planning performance. At this point, I have a couple of doubts: My main concern is that participants do not need to have a complete plan at the end of the planning phase, but that they feel urged by the instructions to end the planning phase without having a fully formed plan. Instead they continue planning or refining their route plan while they are tracing the route. So, an alternative explanation of their findings is that participants only form coarse plans under time pressure, rather than sub-optimal plans, which they refine during the tracing phase.

We agree with this possible description of the planning during this task. Since we consider both aspects of the task route planning and we don’t have the ability to distinguish between coarse or suboptimal plans, we have re-framed the message to better convey the interesting effects of urgency. The effect of urgency on route length (the portion which could involve refining of a plan) was conditional on subjective stress, but it was the same direction as the effect of urgency on planning time (i.e., less planning time in addition to shorter routes traced).

Therefore, we can conclude that in the alternative explanation, even if urgency resulted in more coarse plans in the planning time phase, it also resulted in shorter routes traced during the tracing phase, which could be a result of cognitive processes or fine motor performance. We did not report on the time spent tracing, but the tracing time was positively correlated with route length, so if shorter routes were due to more plan refinement, refinement during the tracing phase might be negatively correlated with tracing time. We did not speculate on this alternative explanation in the manuscript.

This might also explain why routes under time pressure instructions were less efficient than without explicit instructions.

We didn’t test route efficiency, only route length, which does not account for the optimal route length. We did test ideal route length (“as the crow flies”) as a covariate in exploratory analyses, which did not change the conclusions from the confirmatory tests.

I am not convinced that the authors can address this point unless they run additional experiments, as the current procedure does not allow them to assess how refined route plans are at the end of the planning phase.
This is a limitation of the method, but it does not preclude interpretation of the results. We changed the language in the manuscript to better reflect our conceptualization of route planning, which could include plan refinement. The changing of plans during planning or during locomotion is an interesting direction for future research, but we did not highlight that idea in the revised manuscript.

Description of the task and materials: The authors should report in detail how the map stimuli were designed, what criteria they used to design the maps, how many alternative routes between start and destination were (reasonably) available, etc.

We describe the process in more detail in the revised manuscript, and also provide a description here.

The maps were created by taking screenshots of google maps. The location for each map stimulus was relatively random. A graduate student looked for areas that were in or adjacent to cities or towns, such that there were multiple ways to get from place to place. Each map had at least two routes that were roughly equivalent, although one could be slightly shorter. There were also potential cognitive differences (e.g., one route option might have fewer turns). There were often more than two possible routes, but we noted at least two routes while creating the stimuli.

The authors also mention that some map stimuli had additional waypoints. Depending on where the waypoints were positioned, they could either reduce or increase the difficulty of the planning task and affect the number of reasonable alternatives participants need to consider. Overall, the description of the materials and methods should be improved. For example, the authors need to explain how participants were instructed - were they instructed to plan the shortest possible route?
We made sure that the participant instructions were clearly described in the manuscript. Participants were instructed to complete as many routes as possible and motivated by a small performance bonus for each route completed successfully. These instructions could imply that they should plan shorter or more efficient routes, but the explicit instruction was only to complete as many routes as possible, without tracing errors, in the allotted time.

Detailed comments:

The authors refer to their task as a wayfinding task. I believe that the community would expect wayfinding tasks to have an element of actual navigation/movement through space, rather than just planning routes from maps. I would therefore suggest that the authors think about the terminology they use as the address one aspect of wayfinding with maps, i.e. the initial planning stage from maps, but their work cannot speak to how stress might affect the execution of the plan (which is more than just locomotion as it also required memory and decision making to actually follow the route). This is further supported by Lynch’s quote “the process of determining and following a path or route between an origin and a destination”.

We agree that our study is not wayfinding that includes large-scale navigation/movement through space. However, there is an element of behavioral performance in the tracing portion of the task, so we hesitate to draw a hard line between wayfinding in this task and wayfinding in the real world, beyond the admittedly very clear distinction between fine and gross motor performance. To highlight the importance of our task, and also avoid confusion with other wayfinding tasks, we have replaced the word “wayfinding” with the more precision phrase “route planning” when there could be ambiguous interpretations. We also avoided referring to “aided wayfinding”, since that could also imply something other than route planning.

I find it difficult to reconcile H0 and H2 which seem to contradict each other: H0 states that less time would result in fewer trials, while H2 states that less time should result in quicker planning, i.e. shorter trials, which should increase the number of trials. This should be clarified. I assume this has to do with substantially longer waiting times than planning times, however, this would then render H0 trivial.

Time pressure is complicated. There can be a total time limit, which could simultaneously decrease the “amount of task completed” (e.g., the distance travelled or the number of map trials completed), but also increase the speed at which the task is completed (e.g., driving/walking/biking speed, or planning and tracing time). We clarified in the document that planning time in H2 refers to the time between stimulus presentation and the beginning of tracing, which occurs on every trial completed by each participant.

I am not sure if this relates to a requirement of the journal in which case I would suggest to redirect this comment to the editor, but I much prefer having figures embedded in the text. This makes it so much easier for reviewers to read and assess the paper as there is no need to endlessly scroll back and forth through the document.

This is a requirement of the journal.

Do you have any information about the interaction device participants used to complete the task. I would imagine that completing the task with a mouse or trackpad could substantially affect the times required to trace a route.

We programmed the experiment to detect touchscreen use (to increase confidence in exclusions for touchscreen use) and participants also self-reported device use. Based on this feedback, we tested self-reported trackpad compared to mouse use as a covariate in exploratory models, reported in S1_File, Table S5. The difference was not statistically significant (H1-H3) unless the models controlled for subjective stress (H4-H5). Including the covariate did not alter conclusions and we mentioned this in the manuscript.

I am unclear what the following text means: “A map stimulus was presented at the start of each trial. The cursor appeared as a blue dot when ‘s’ was pressed to signal the end of the planning phase. The screen above was after a route was traced, but before ‘f’ was pressed to signal that the route was complete.”

We revised the description to avoid unnecessary detail like the appearance of the cursor as a blue dot while conveying the relevant information about the trial procedure.

Fig 3 suggests that it may take some time to find the start and destination location which were only two symbols among a lot of additional markers in the maps and it is unclear what a waypoint is (referred to in the text but not explained in detail). The screenshots of the maps stimuli are of low image quality which makes it difficult to read the maps and therefore understand the task. Was the time to find the start location, the destination and potential waypoints included in the planning phase?

We revised the manuscript to convey that location identification is conceptually part of the planning time, but a participant could have ended the planning phase and began the tracing phase without locating all the points. We also removed screenshots of the maps seen by participants from the publication due to licensing restrictions, while providing them for fair use in the online repository.

The authors state that the planning phase is distinct from the tracing phase. What prevents participants from moving into the tracing phase without having completed the plan and using time in the tracing phase to complete the planning process (i.e. while planning)? I believe this is an important issue to address as some route planning literature suggests that routes unfold during navigation (even though participants are not actually navigating in this experiment). In any case, I could imagine that participant who are put under time pressure might be tempted to start tracing the route without having a complete plan. Instead they could start tracing roughly in the direction of the destination and completing the plan as they trace (which might also explain longer trajectories under time pressure).
This is related to one of the major concerns addressed above and we generally agree that this is a plausible description of behavior during the task. However, the data suggest that if urgency messaging affects route length (see H3 and H5), it would result in shorter trajectories – although this effect was only present when subjective stress reported after the trial was included in the model; subjective stress was associated with longer routes (H5).

Figure caption seem to be missing or they are very short. I would have found it helpful to have more informative figure captions explaining the figures in more detail. For example, Fig3 depicts numbers above the map stimuli and the meaning f these numbers were first explained in the Procedure section whereas I believe it should be presented where the materials are presented and in the Figure caption.
We revised the figure captions.

“The first between-subjects manipulation was a countdown timer ...” -> I think it would be more precise to say that the time was the manipulation, as both conditions had a timer (if I understand correctly).

We revised this statement.

A description of the map stimuli or the route planning tasks is missing. How were these maps designed, what were the criteria for designing the route planning tasks? How were waypoints introduced. How many alternative paths were possible, how were the maps selected for each participants, how did the authors ensure that participants in the different conditions had comparable planning tasks, etc. All this is crucial information which seems to be missing from the manuscript and which needs to be explained in detail.

We added the relevant information to the manuscript.

“The routes traced averaged 1.86 “height units””. It is unclear what that means. The authors should explain exactly how they calculated the route length, how the shortest possible route was determined and how they calculated participant performance. Route planning performance should ideally be described as a percentage above optimal or using a similar measure.

The route length was not calculated relative to an optimal route in confirmatory analyses. We focused on two aspects of performance involved with route planning, 1) how long would people take to plan (including BOTH point identification and path planning) before starting to trace, and 2) what is the two-dimensional distance of the line drawn by people tracing a route. There are many other research questions that could be asked about the efficiency of the tracing or the length of the traced route compared to an optimal route, but those questions were not the focus of this investigation.

In addition to using random intercepts to account for the differences in possible route length between stimuli, we tested ideal route length as a covariate in exploratory models, which did not change any conclusions pertaining to the confirmatory hypothesis tests.

What exactly were the instructions that participants received. Were they instructed to plan the shortest possible route?

This is one of the major concerns that we addressed above. Participants were instructed and motivated to complete as many trials as possible within the allotted time.

Do the authors think that the requirement to trace the routes could impact participants planning strategies. Could they, for example, prefer longer routes that are easier to trace, as they have fewer turns? This should be addressed in the revision.

We do think that the tracing is important. In fact, many aspects of fine motor performance are interesting to think about, but not directly relevant to the primary research questions in this manuscript. Systematic differences related to both environmental features and individuals have been considered statistically in the model structure (i.e., random effects for stimuli and participants) used for confirmatory hypothesis testing. We have made the performance data available, along with the stimuli, for anyone interested in pursing additional research questions.

Figure 7 shows that mean planning time is far higher than median planning time suggesting that outliers strongly affect the data. Have the authors conducted outlier analyses? If not, I would suggest to do so and to rerun the analyses.

The models with planning time as an outcome did not assume a normal (Gaussian) distribution and we excluded trials for confirmatory hypothesis testing based on the preregistered criteria.

How did planning time differ for trials with and without waypoints?

The models accounted for differences in each outcome that were due to the stimulus, so the main conclusions should be robust to any differences that were due to the stimulus characteristics. We confirmed this assumption and documented differences associated with map type in exploratory models that are reported in the Supplemental Material.

The strength and limitations and the future research sections in the discussion are almost half of the discussion. I believe this is too long and is something I would expect in a thesis rather than a journal publication. I would suggest to shorten these sections.

We tightened up the strengths, limitations, and future directions.

**Reviewer #2:**

This paper examined the contributions of three aspects of time pressure, total amount of time allocated to complete a task, the length of wait times interspersed between trials, and instructions to hurry up. Results indicated that only instructions to hurry up (urgency) increased subjective ratings of stress at the trial level. The results were somewhat contradictory in that urgency decreased planning time and route length and also increased subjective stress, but subjective stress was associated with both increased planning time and longer routes. The authors speculate that subjective stress may have reflected participants self-assessments of their performance, rather than the stress they felt at the beginning of the trial when told to hurry up. The goal of examining the effects of different aspects of time pressure on navigation is a good one, this is a solid, well conducted experiment, and the results are appropriately analyzed and interpreted.

However I have several reactions that I feel could be addressed a revision to improve the contribution of this paper. Specifically, I am concerned that two of the manipulations would not have been expected, a priori, to impose time pressure (at least if I am understanding them correctly), so it is not surprising that they did not, and I also question the ecological validity of the task. In addition, I had some questions about the methodology and it was hard to integrate the text and figures.. Finally the general discussion seems overly concerned with the limitation of the study and future directions, rather than arguing for the contributions of this research.

Thank you. We believe that we have addressed each of the concerns in the revised manuscript.

First, it is not clear to me how the total time manipulation is a time pressure manipulation at all, as it seems that what was varied was the total amount of time that people had to do a number of route planning trials, but there was no indication of how many trials they had to do in this time, and it seems that at a trial level they were told to take their time. Imposing a time limit per trial would have been a better manipulation (and maybe this was done but that is not what I understood from what I read)

Imposing a time limit on every trial is a traditional way to operationalize time pressure. In this case, we changed the time limit on the whole task. We hoped that participants were motivated to complete as many trials as possible by the very small reward that we offered on a per-trial basis, along with any general motivation to follow the instructions and complete as many maps as possible in the allotted time. Allotting less time, in total, may have caused participants to work more quickly, planning faster, tracing faster, and completing more trials. Instead, what we saw is that participants completed fewer trials when they had less time to work on the task. This finding, along with the manipulation check, suggests that altering the total amount of time allotted did not generate time pressure. It is of course reasonable that other scientists may have generated different hypotheses a priori, or conducted a different experiment.

Second, I could not find any information on whether the wait times were within trials or between trials. I think they would be more likely to induce stress if they were within trials, so if they were between trials again I would not have thought, a priori, that this would have induced time pressure or stress.

We clarified that the waiting periods occurred between trials. We thought that because the waiting periods deducted from the total allotted time, it was possible that the waiting periods would have caused time pressure. Again, we found it interesting that when we reduced the total amount of time available to complete the task, it did not elicit time pressure, even though other people may have hypothesized that a priori.

Third, the task of planning and then tracing a route on a map does not seem very ecologically valid, as it does not include the locomotion aspect of wayfinding, that is, the experience of navigating under time pressure in the real world, something that the authors themselves admit in the general discussion.

This was also mentioned by Reviewer #1, so we refer to the responses above. In particular, we have clarified that we are focused on route planning.

Fourth, as noted above, I had work hard to understand the tables and figures, as the variable and column names in the figure (e.g.. low/high, low/low) were not explained in the captions (I figured them out but had to work at it) and the figures were hard to understand because all figures were at the end of the manuscript, while their figure captions appeared in the manuscript itself, and the figures at the end were not labelled by number.

We revised the figure captions, but the formatting of the figures appearing at the end, separate from their captions, is part of the journal formatting and not under control of the authors.

The authors motivation could also be strengthened. It seemed to come down to “nobody has done this before”, but not every study that has not been done should be done, so that is not sufficient justification. Specifically, he introduction could be improved by some more theoretical or at least practical motivation for why different aspects of time pressure might have different effects, to motivate why this research is important.

We thought that different operationalizations of time pressure might have similar effects, rather than different effects, which is noted in the preregistration and in the manuscript. Using multiple time pressure manipulations allowed us to determine which, if any, of the operationalizations were effective at inducing time pressure and subjective stress.

Similarly much of the general discussion was devoted to a list of limitations of the present study, as if the authors were writing a negative review of their own paper. For example the broad sample used is a strength of the paper (much better than the typical college sample) but the authors seem to almost interpret it a limitation by questioning whether it truly represents the population. Similarly the section on future possible studies seems too long (this is a journal article, not a grant proposal) so i suggest they rewrite (and shorten) the general discussion to emphasize what they learned and the strengths of the present study, rather than the limitations, and focus on more specific future studies that would help explain the results of this one.
We revised the discussion, highlighting the advances made and tightening up the strengths, limitations, and future directions. We proposed one specific future direction to help explain the null results of the total time and wait time manipulations, which is to design those manipulations as within-subjects factors at the trial level.

Specific comments:
Page 3? “Wayfinding was coined …” -> should be “the term wayfinding was coined”

We corrected this statement.

Page 10: I was unable to parse this sentence: “The screen above was after a route was traced, but before ‘f’ was pressed to signal that the route was complete.”

We revised the description of the task instructions and procedures.

The graphs of route time indicated that it was very skewed. Some outlier analysis or a log transform might be appropriate.

The models with planning time as an outcome did not assume a normal (Gaussian) distribution and we excluded trials for confirmatory hypothesis testing based on the preregistered criteria.

The language in the paper is sometimes more conversational than is appropriate for a journal article, for example shortening do not to “don’t” and although to “though”

We changed every instance of “though” to “although” and every instance of “don’t” to “do not”.
