# Supplementary material for: How much time to figure out how to get where? Route planning and subjective stress under time pressure
Source: PLoS One. 2025 Jan 13;20(1):e0316382. doi: 10.1371/journal.pone.0316382 (PMC11729951; doi:10.1371/journal.pone.0316382)
Supplement: Attachment — Submitted filename: Response to Reviewers.docx [file pone.0316382.s005.docx]

**Reviewer #1**: The authors have addressed most of my original comments.

Figures: I cannot find the figures in the revised manuscript which makes it difficult to assess several of the revisions.

We are not sure why the documentation provided to the reviewer seemed not to contain the figures. The figures are not embedded in the manuscript, per the journal requirements, but they were visible to us, starting on p. 54 of the manuscript pdf generated by the submission portal.

Map Stimuli: While the process of generating the maps has improved in the revision, I believe the rationale for having three categories or sets of maps should be further detailed. In addition, I would suggest to present at least one examples for each map type (see comment above about Figures not showing in the submitted revision)

We created multiple categories of map, based on the placement of the points, because we wanted to be sure there was variation in the kinds of routes participants had to plan and trace. We improved the description of this rationale in Materials/Map Stimuli.

Map type was not a variable we intended to focus on as a manipulated factor in analyses. We did, however, explore the type of map in the Supplemental Material. Adding the map type as a covariate did not change our confirmatory results, but there were statistically significant, yet unsurprising differences in the performance metrics based on map type.

All map stimuli are available at <https://osf.io/w36xe/>. We uploaded the .PNG files separately so that one does not need to download the entire task folder to access any single map image. The map stimuli themselves are not included in the document due to copyright restrictions. The file that shows the coordinates (S1_Map_Coordinates.csv) has been updated to include a column showing which of the three types of map each stimulus represents.

Methods: I cannot find information about how the maps were presented to participants. Were maps randomly assigned to the different conditions or was this balanced between participants?

For each participant, one map was randomly assigned at the beginning of each trial, without replacement and without regard for any between- or within-persons condition. This has been clarified in the Procedure (L407-409).

Route Planning Task & Motor Performance: I am still a little bit puzzled about the planning task, especially related to the tracing aspect and the required motor performance. Why was the decision made to ask participants to trace the chosen/planned path. How does that aspect of the task relate to the real world examples, the authors introduce?

The tracing part of the task enabled us to assess route length as one aspect of motor performance. In general, we acknowledge that this laboratory-style task is, of course, different in lots of ways from real-world examples. This new procedure nonetheless leaves future research to explore how the route length outcome from this task is associated with characteristics, including lengths, of routes planned or navigated in real-world situations.

Route Planning Task & Motor Performance: I understand that motor performance is not a focus on the study, but I assume that time pressure also affected motor performance in the tracing task. Is this true? How many tracing errors were there and how many trials were discarded because participants did not meet the motor performance expectations?

We think of route length as one indicator of fine motor performance because, while the route length is highly determined by the stimulus (and this is accounted for in the models), there may be some variability in route length related to the ability of participants to complete the task with their hand. The two-dimensional route length was the only outcome that involved fine motor performance we conceptualized in our preregistration for this study, including the hypotheses and analysis plan. However, like the reviewer, we are very interested in other outcomes. In fact, we are currently pursuing publication of a separate manuscript that examines a wider range of motor performance outcomes based on data collected in additional studies.

We clarified the exclusion procedure in this revision, to better convey that exclusions took place at the participant level first. Based on the pre-registered performance criteria, 19% of the participants were excluded because, on at least five trials, the data indicated that they either 1) did not click the right-hand button to start the trial, 2) had a planning time greater than 60 seconds, or 3) did not attempt to follow the paths and draw a route through all the marked points. Only 18 trials (<1%) were excluded due to the performance review flag, after the participant-level exclusions.

Route Planning Task & Motor Performance: How long, on average and for the different conditions, did it take participants to trace the routes? Overall, I find it surprising that the tracing aspect and the motor skills required to trace the routes is such an important part of the task but is not addressed in the analysis at all.

In this manuscript, we chose to focus on the pre-registered, confirmatory hypotheses. However, we have added an additional exploratory model to the main text that uses the outcome of “tracing time”. We expected that tracing time (*M* = 35.93 s) would be correlated with route length, so much so that we thought it may be redundant (i.e., if a route is longer, it should take longer to trace that route). Although the correlation was large, *r* = .56, it was not as large as we expected *a priori*. Tracing time results were added to Tables 2-4 in the main text.

The distribution of the tracing time metric was similar to the planning time metric, so we modelled it as a non-normally-distributed outcome, using a generalized linear mixed-effect model with a gamma distribution and a log link. The exploratory model with the tracing time outcome provides additional information that should supplement the pre-registered performance outcomes (i.e., planning time, route length) that we conceptualized for confirmatory hypothesis testing.

In their response to reviewer comments the authors argue that the search for the start/destination/waypoints is part of the planning process. It would be good to see more example stimuli to develop a better understanding of how long it might take to find these symbols to get a better idea of the relative impact of the different processes (search, actual path planning) on overall planning time.

As mentioned above, all map stimuli are available at <https://osf.io/w36xe/>.

In their response to reviewer comments about outliers in the plan time analysis, the authors respond that they “excluded trials for confirmatory hypothesis testing based on the preregistered criteria.”. I am not sure what that means. Have they actually excluded outliers? I believe this is an important point as the data looks very skewed.

We did not plan to exclude outliers based on a statistical cutoff like a z-score. Instead, we carefully considered the conditions before data collection and made *a priori* decisions about the conditions under which the data would be invalid (e.g., a planning time greater than 60 seconds). When the planning time outcome was not normally distributed, we changed our analysis plan and applied a gamma distribution with a log link to address that specific violation of distributional assumptions when modeling planning time (see Results, Assumption Check).

We excluded one participant and one trial for unexpected reasons that were clearly justifiable (one participant essentially did not complete the task, so had one extraordinarily long planning time, and, for another participant, one trial had a planning time that was erroneously zero). Although it may be tempting to test outlier exclusion procedures, it is important to have a valid reason for doing so, because removing outliers only to improve model fit could lead to invalid conclusions. However, to address the reviewer’s point directly, we conducted a test of influential observations for all models reported in the main text. We examined Cook’s distance as an arbitrary metric that indexes the change in model estimates when observations are excluded from the model, one by one.

When we re-ran the models excluding observations with a Cook’s distance < .5, the results did not alter our main conclusions about urgency messaging and subjective stress. The statistical significance of three fixed-effect predictors was affected. The changes in statistical significance were only observed in the generalized linear mixed-effects models that used non-normal distributions, for which the Cook’s distance may not make sense. After excluding 2 influential observations in the model for H4, the estimated effect of long waits compared to short waits was no longer statistically significant (*OR* = 1.115, *p* = .030 to *OR* = 1.133, *p* = .159). After excluding 3 influential observations in the model for H4E, the estimated effect of long waits compared to short waits was no longer statistically significant (*OR* = 1.101, *p* = .001 to *OR* = 1.110, *p* = .173) and neither was the association between subjective stress at the individual-level and tracing time (*b* = 0.001, *p* < .001 to *b* = 0.003, *p* = .096).

Route length: I do understand that the authors analyse route lengths but I am not sure I understand what exactly participants were instructed to do. Were they instructed to plan short routes (I assume this is the case otherwise it is unclear why the authors chose the route length as a DV). If the authors explicitly instructed participants to plan short routes, why was the chosen route not compared to the optimal route which seems the most logic measure (unless of course, participants planned routes that were easy to trace - see point above)? In their response to a similar earlier comment, the authors just stated that they chose two-dimensional distance and that other measures could be looked at but they do not motivate why they choose distance and what it tells us about the route planning process.

Thank you for giving us the opportunity to clarify. We shared our reasoning regarding a focus on route length on lines 172-176, where we said, "Finally, because time pressure generally increases speed and decreases accuracy we expected that the higher-time pressure conditions would result in longer routes than the lower-time pressure conditions (H3). However, because the effects of acute time pressure in new map environments have not been studied extensively, we intentionally remained open to opposite or null effects of all three experimental manipulations.” We note on lines 286-287 that "Instructions emphasized tracing as many routes as possible without making tracing errors."

In general, we expected that the two outcomes we selected would provide useful information about performance in this task, which was designed as a controlled laboratory-style experiment that would allow participants to plan routes in previously unlearned environments. We expected the performance metrics to be affected by the manipulated temporal conditions. However, we do not claim that this task directly assesses how participants plan routes in real life.

Route length & Route tracing: Related to the points raised above, I would be interested to know if the authors agree that the specific procedure and the requirement to precisely trace the chosen routes could influence route choice? Without having performed the task myself, I suspect that tracing a route closely is difficult. If so, this requirement could well affect route choice, such as choosing the simplest route. If that was the case, the results are strongly affected by the response mode, and one could question what the results tell us about route planning itself.

Agreed, the task enables participants to make route choices. It's entirely plausible that route choices are impacting planning time and route length outcomes. The rational move under time pressure is to indeed try to draw simple routes so they take less time to plan and trace. This will definitely be useful to explore in future studies. Interestingly, only about 9% of variation in route lengths is due to participants whereas a whopping 69% of variation in route lengths is due to the different map stimuli. So the routes people trace are largely due to features of the maps themselves; some maps require longer routes than others.

Analysis: Why was map set (no waypoints, waypoints, round trip) not included in the analysis as a factor. This relates to an earlier comment about the missing motivation for including the different map types.

We included the map type as a predictor in the models reported in the Supplemental Material. The interpretation of the models used for confirmatory hypothesis testing did not change when we accounted for the type of map; however, map type was associated with some outcomes. Furthermore, in the current revision, we included both map type and variation in the length of the ideal routes (i.e., as the crow flies) and the interpretation of the models used for confirmatory hypothesis testing did not change. However, ideal route length was also associated with some performance outcomes.
